# Embryo‐derived teratoma *in vitro* biological system reveals antitumor and embryotoxic activity of valproate

**DOI:** 10.1111/febs.15248

**Published:** 2020-02-28

**Authors:** Milvija Plazibat, Ana Katušić Bojanac, Marta Himerleich Perić, Ozren Gamulin, Mario Rašić, Vedran Radonić, Marko Škrabić, Maria Krajačić, Jure Krasić, Nino Sinčić, Gordana Jurić‐Lekić, Maja Balarin, Floriana Bulić‐Jakuš

**Affiliations:** ^1^ Department of Pediatrics Hospital Zabok Croatia; ^2^ Centre of Excellence for Reproductive and Regenerative Medicine Unit for Biomedical Investigation of Reproduction and Development School of Medicine University of Zagreb Croatia; ^3^ Dental Medicine and Health School of Medicine University of Osijek Croatia; ^4^ Department of Medical Biology School of Medicine University of Zagreb Croatia; ^5^ Department of Physics School of Medicine University of Zagreb Croatia; ^6^ Center of Excellence for Advanced Materials and Sensing Devices Research Unit New Functional Materials, School of Medicine, University of Zagreb Croatia; ^7^ Department of Head and Neck Surgery Tumor Clinic,Clinical Hospital Center Sisters of Charity Zagreb Croatia; ^8^ Department Of Cardiology Clinical Hospital Merkur Zagreb Croatia; ^9^ Department of Histology and Embryology School of Medicine University of Zagreb Croatia

**Keywords:** histone deacetylase inhibitor, IR spectroscopy, teratoma, valproate

## Abstract

Antiepileptic/teratogen valproate (VPA) is a histone deacetylase inhibitor/epigenetic drug proposed for the antitumor therapy where it is generally crucial to target poorly or undifferentiated cells to prevent a recurrence. Transplanted rodent gastrulating embryos‐proper (primitive streak and three germ layers) are the source of teratoma/teratocarcinoma tumors. Human primitive‐streak remnants develop sacrococcygeal teratomas that may recur even when benign (well differentiated). To screen for unknown VPA impact on teratoma‐type tumors, we used original 2‐week embryo‐derived teratoma *in vitro* biological system completed by a spent media metabolome analysis. Gastrulating 9.5‐day‐old rat embryos‐proper were cultivated in Eagle's minimal essential medium (MEM) with 50% rat serum (controls) or with the addition of 2 mm VPA. Spent media metabolomes were analyzed by FTIR. Compared to controls, VPA acetylated histones; significantly diminished overall teratoma growth, impaired survival, increased the apoptotic index, and decreased proliferation index and incidence of differentiated tissues (e.g., neural tissue). Control teratomas continued to grow and differentiate for 14 days in isotransplants *in vivo*, but *in vitro* VPA‐treated teratomas resorbed. Principal component analysis of FTIR results showed that spent media metabolomes formed well‐separated clusters reflecting the treatment and day of cultivation. In metabolomes of VPA‐treated teratomas, we found elevation of previously described histone acetylation biomarkers [amide I α‐helix and A(CH_3_)/A(CH_2_)]) with apoptotic biomarkers within the amide I region for β‐sheets, and unordered and CH_2_ vibrations of lipids. VPA may be proposed for therapy of the undifferentiated component of teratoma tumors and this biological system completed by metabolome analysis, for a faster dual screening of antitumor/embryotoxic agents.

## Graphical abstract

Valproate (VPA; antiepileptic/teratogen/histone deacetylase inhibitor) negatively affected embryo‐derived teratoma development *in vitro* and abolished its potential to recover *in vivo*. FTIR spectroscopy results separated all VPA‐treated and control metabolomes, while FTIR biomarkers reflected processes of apoptosis and histone acetylation assessed in teratomas by immunohistochemistry and western blotting. We propose this *in vitro* biological system, completed by analysis of spent media metabolomes, as a screening system for embryotoxic and antitumor agents.

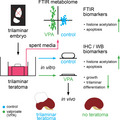

**Abbreviations**
ASD: autism spectrum disorder
EC: embryonal carcinoma
GTS: growing teratoma syndrome
HDACi: histone deacetylase inhibitor
MEM: minimal essential medium
PCA: principal component analysis
PCNA: proliferating cell nuclear antigen
RMSEC: root mean square error of calibration
RMSECV: root mean square error of cross‐validation
SAHA: suberoylanilidehydroxamic acid
VPA: valproate
WEC: whole rat embryo

## Introduction

Valproate (VPA) is an antiepileptic/teratogenic drug from the WHO Model List of Essential Medicines [1] that may cause fetal VPA syndrome in children whose mothers were treated during pregnancy [2, 3]. Some results of *in utero* exposure to VPA correlate with autism spectrum disorder (ASD), while postnatal treatment can ameliorate ASD [4], outlining the difference between VPA activity in a developing and the adult organism.

Valproate is a histone deacetylase inhibitor (HDACi) [5, 6], an epigenetic drug that changes gene expression, which may affect both the normal or aberrant developmental programs (teratogenesis or tumorigenesis, respectively). Based on preclinical research, some HDACis are already in clinical use for therapy of lymphomas and multiple myeloma [7], while VPA itself has been used in preclinical and clinical settings for breast cancer adjunct therapy [8]. Basic research on the possible VPA therapeutic activity for other solid tumors is still ongoing [9, 10]. To our knowledge, teratoma‐type tumors have not yet been treated by VPA, and this *in vitro* research is the first attempt of screening for therapeutic influence of VPA on a teratoma.

By definition, teratoma is a benign tumor because it consists of well or somewhat less differentiated tissue derivatives that denotes a slow‐growing, low‐grade tumor [11, 12]. However, even teratomas that were histopathologically diagnosed as benign may recure. The best example is human sacrococcygeal teratoma that seemingly arises from the remnants of the primitive streak [13, 14]. The primitive streak forms in the epiblast (primitive or primary ectoderm) and marks the beginning of gastrulation. During gastrulation, active cell migration through the primitive streak is necessary for the formation of definitive embryonic germ layers (ectoderm, mesoderm, and endoderm) that are the source of differentiated tissues in various organs [15]. Teratoma is usually trilaminar, meaning that it consists of derivatives of all three germ layers [16]. Importantly, for the appropriate therapy of various tumors that always depends on histopathological diagnosis, histopathological classification based on developmental histogenesis (ectodermal, mesodermal, or endodermal origin of tumors) is consistent with modern molecular analysis of tumors [17].

Teratoma can experimentally be obtained by *in vivo* isotransplantation of the rat gastrulating embryo‐proper, while isotransplantation of the mouse gastrulating embryo‐proper leads to the development of teratocarcinoma, additionally containing undifferentiated malignant embryonal carcinoma (EC) cells [18]. In humans, teratomas/ECs may be diagnosed in the testes [19], while teratomas diagnosed in ovaries are usually benign, although they may be malignantly transformed even after a decade from the first diagnosis [20, 21]. In the rare growing teratoma syndrome (GTS), histopathologically assessed benign teratomas that also lack serum markers of malignancy may recure several times in various sites with the incidence from 1.9% to 7.6% during or after chemotherapy in patients with nonseminomatous germ cell tumors of the testis or ovary [22].

In the established embryo‐derived teratoma biological system *in vitro*, we have been able to screen for the specific activity of various extraneous agents or culture conditions [12]. Among drugs we tested so far was the DNA hypomethylating agent 5‐azacytidine that is used for the epigenetic therapy of myelodysplastic syndrome [23, 24] and retinoic acid [25] used for the differentiation therapy of leukemia [26] or widespread acne vulgaris [27]. Moreover, we have recently described specific hyperthermal regimes able to destroy experimental teratoma tumors or enhance their differentiation *in vitro* [28].

The source of the embryo‐derived teratoma biological system is the gastrulating embryo‐proper with the primitive streak forming three germ layers, devoid of extraembryonic parts. After isolation, the architecture of the postimplantation embryo is lost and the three germ layers, containing only stem cells of the three basic cell lineages (ectodermal, mesodermal, and endodermal), immediately start to develop a disorganized mixture of differentiated tissues (epidermis, neural tissue, cartilage, muscle, and derivatives of the primitive gut epithelium) that are usually assessed at the end of the 2‐week culture period. The resulting structure resembles a trilaminar teratoma solid tumor. Differentiation of tissues is possible only at the air–liquid interface in the optimal rat serum‐supplemented medium, while submerged embryos‐proper do not differentiate into a teratoma [29, 30]. In the natural three‐dimensional (3D) system, where tissue interactions are not as disrupted as they are during the establishment of two‐dimensional (2D) cell cultures [31], a parallel assessment of the survival, overall growth, and differentiation is conducted [28].

As shown previously, by subsequent isotransplantation of the precultivated embryo‐derived teratoma to the metabolically richer environment *in vivo* for another 14 days, the residual potential for further growth or differentiation (e.g., of skin appendages, bone, neural tissue, and even organotypic structures) or the restriction of such potential was assessed [32, 33]. A similar teratoma assay in rodents *in vivo* is used to confirm pluripotency [34] or discover a residual potential for malignant transformation of various cells precultivated *in vitro* for regenerative medicine purposes [12, 35].

Although animal *in vivo* testing in rodents is still the way to find new treatments for human diseases, especially for the complex, multifactorial diseases such as cancer, as well as for the assessment of reproductive and developmental toxicity, pharmaceutical toxicologists have been lately interested in predicting toxicity using simple, inexpensive methods. Employment of *in vitro* biological systems tends to reduce the number of living animals in experiments according to the replacement, reduction, and refinement (3Rs) rule of Russel and Burch [36, 37, 38, 39]. Because the embryo‐derived teratoma system starts with the critical developmental stage of gastrulation when the ‘all or none’ rule is lost from embryos and a therapy may result in congenital anomalies [12, 40, 41], this system also reveals the developmental toxicity of embryotoxic/teratogenic drugs or physical factors [24, 25, 28].

As a proper biological system should include analysis of all of its components [42, 43], we completed the used system conducting parallelly metabolome analyses of spent culture media with FTIR spectroscopy followed by multivariate data processing [44, 45], similarly as was used for prediction of the preimplantation embryo quality [46]. FTIR spectroscopy is detecting vibrations of chemical and functional groups of molecules within complex biological samples to obtain a spectral fingerprint and was proposed for the fast analysis of biofluids, allowing the subsequent classification of spectra from different categories with computational methods and possibly the identification of biomarkers [47]. The advantages of this label‐free approach are the fast analysis of a large number of samples from minimum material, and a low cost [47, 48, 49, 50].

We now report the impact of the VPA on the rat embryo‐derived teratoma system *in vitro* completed by an analysis of the spent culture media metabolomes by FTIR. Results from the FTIR spectroscopy, especially those about biomarkers of histone acetylation and apoptosis, corroborate the data we obtained regarding the negative impact of VPA on the teratoma development, thus contributing to a faster screening for antitumor and embryotoxic/teratogenic agents.

## Results

### Teratomas

#### Valproate impairs survival of embryo‐derived teratomas *in vitro*

After 2 weeks, histological analysis showed that 81.1% of the explanted embryos from the untreated control group developed teratomas, which was significantly higher than 59.4% from the group treated with 2 mm VPA. Therefore, VPA impaired survival of embryo‐derived teratomas cultivated *in vitro* (Table 1).

**Table 1:** Survival of embryo‐derived teratomas cultivated with VPA. After the 2‐week culture, survival was assessed by classical histology. The number of teratomas that developed with the addition of 2 mm VPA was significantly smaller than the number of control teratomas (*P* = 0.029).

Cultivation media	MEM + serum		MEM + serum + 2 mm VPA	
	*N*	%	*N*	%
Explanted embryos	37	100	38	100
Teratomas developed *in vitro*	30	81.1	22[^a^	59.4

a ^a^Chi‐square test: *P* = 0.029.

#### Overall growth during a 2‐week culture is negatively affected by valproate

**Figure 1:**
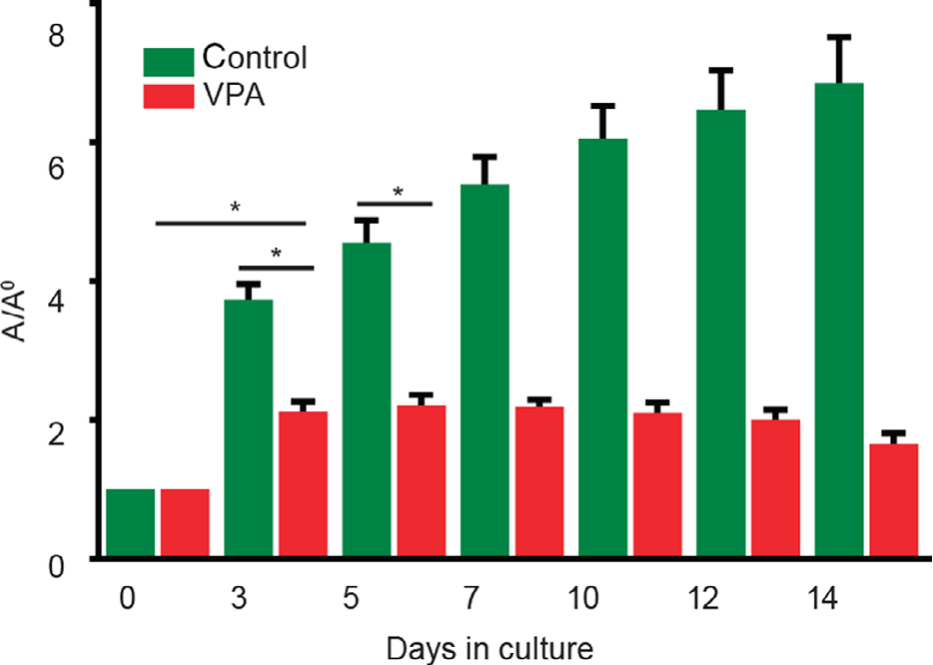
Overall growth of embryo‐derived teratomas with VPA. As the measure of overall growth, calculated ellipse area of teratomas was normalized to the day 0 of plating (*A*/*A*_0_) and was always significantly smaller in VPA‐treated embryo‐derived teratomas (*n* = 37) than in controls (*n* = 38). Mean ± SEM. *P = 0.0001 (Mann–Whitney test).

We explored the impact of the VPA upon overall growth during the 2‐week culture period in comparison with the untreated controls. Both groups of embryo‐derived teratomas cultivated either without or with VPA (2 mm) grew significantly until the 3rd day of culture (*P *˂ 0.0001), although those treated with VPA were significantly smaller than untreated (*P *˂ 0.0001). Therefore, VPA significantly diminished the growth of teratomas already at the beginning of culture in comparison with the controls. Teratomas treated with VPA were not growing beyond the 3rd day of culture in contrast to the controls (Fig. 1).

#### Valproate enhances apoptosis in embryo‐derived teratomas *in vitro*

**Figure 2:**
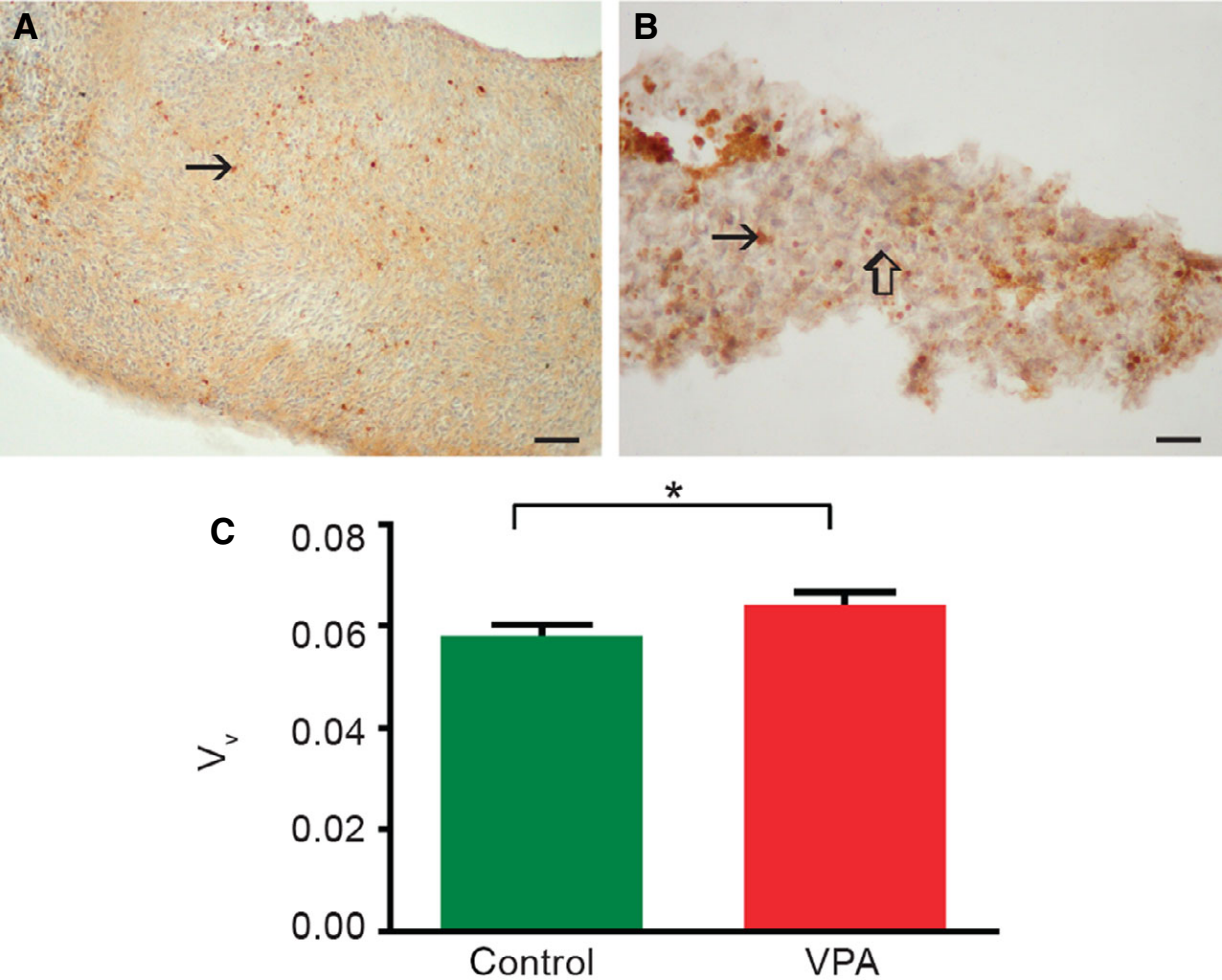
Apoptosis in embryo‐derived teratomas cultivated for 3 days. (A, B) Representative micrographs of cleaved caspase‐3‐positive cells (arrow) in control (A) and 2 mm VPA‐treated (B) embryo‐derived teratomas. Internal negative control (thick arrow). Scale bars: A 50 μm, B 25 μm. IHC, DAB, counterstained by hematoxylin. (C) Stereological quantification of cleaved caspase‐3‐positive cells. *n* = 14 control embryo‐derived teratomas, *n* = 11 VPA‐treated embryo‐derived teratomas. Mean ± SEM *P = 0.0472 (Mann–Whitney test).

To investigate whether VPA diminished overall growth of embryo‐derived teratomas through apoptosis, we assessed the expression of an apoptotic marker (cleaved caspase‐3) by immunohistochemistry (IHC) at the 3rd day of culture when the difference in growth was first assessed. Although apoptosis was detected in all samples by light microscopy (Fig. 2A,B), stereological quantification of the cleaved caspase‐3 revealed that its volume density (*V*_v_) was significantly higher in teratomas treated with VPA than in controls (*P* = 0.0472; Fig. 2C), and we may conclude that apoptosis negatively influenced growth of embryo‐derived teratomas.

#### Valproate negatively affects the incidence of tissues in embryo‐derived teratomas *in vitro*

**Figure 3:**
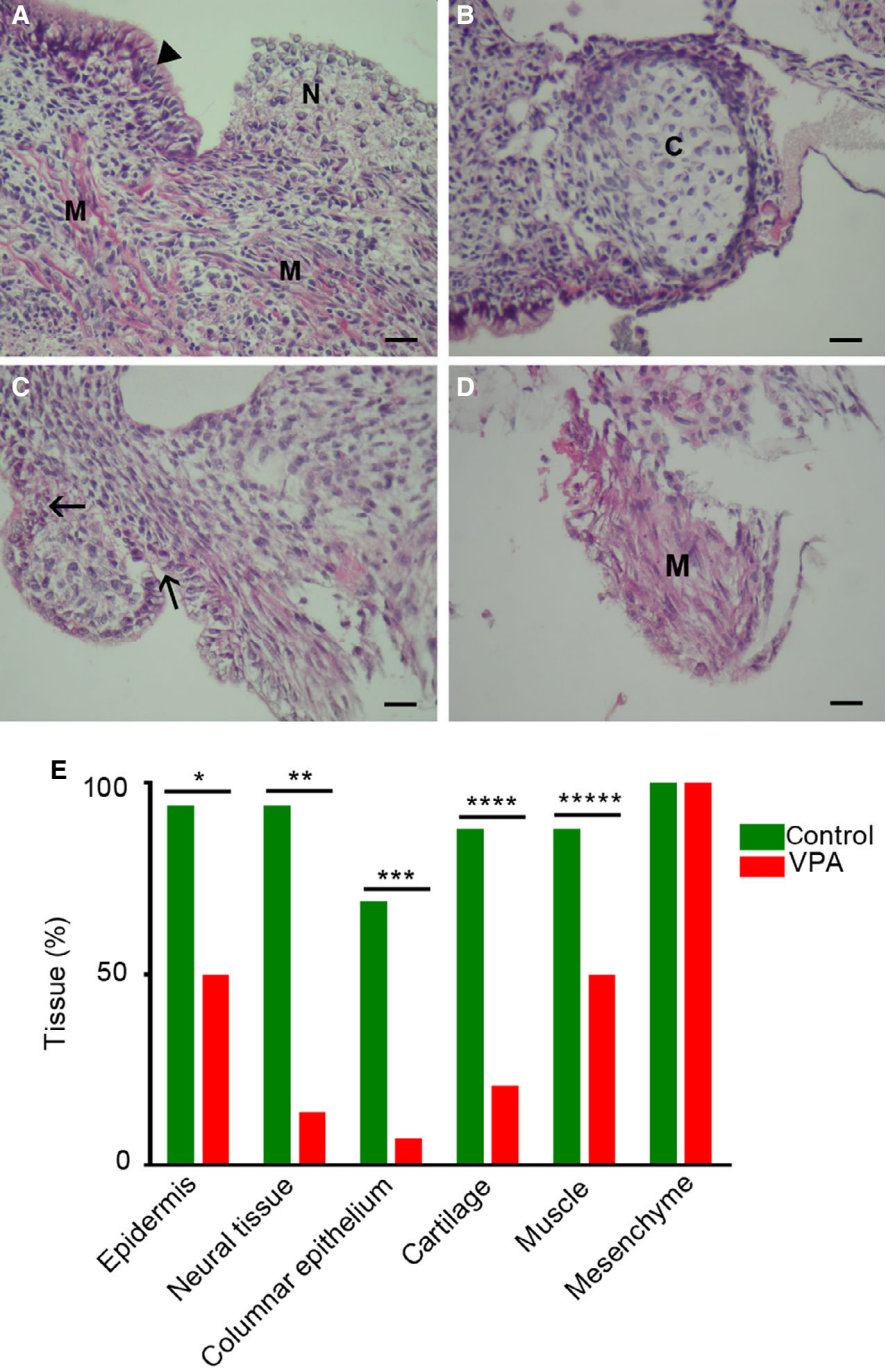
Differentiation in embryo‐derived teratomas cultivated for 14 days *in vitro*. (A, B) Control trilaminar embryo‐derived teratomas cultivated in Eagle's MEM and 50% rat serum. Pseudostratified columnar epithelium (arrowhead); neural tissue (N); muscle (M), cartilage (C), scale bar = 25 μm, hematoxylin–eosin. (C, D) Embryo‐derived teratomas cultivated in Eagle's MEM and 50% rat serum with 2 mm VPA. Cylindrical epithelium (arrows); muscle (M), scale bar = 25 μm, hematoxylin–eosin. E Incidence of tissues (%) in the group of control embryo‐derived teratomas (*n* = 16) and in the group of VPA‐treated embryo‐derived teratomas (*n* = 14). Fisher's exact test, with two‐tailed *P* calculation. *P = 0.0121, **P = 0.0001, ***P = 0.0008, ****P = 0.0006, *****P = 0.0457.

Differentiation in the embryo‐derived teratoma biological system has been described from the second day of culture, and it proceeds toward the end of the culture when tissues are fully differentiated and easily discerned by light microscopy [29]. Accordingly, we histologically assessed and compared the incidence of various differentiated tissues in the controls and VPA‐treated at the end of the 2‐week culture period. In well‐developed controls that were large and contained almost no necrosis (Fig. 3A,B), we assessed a high incidence of differentiated ectodermal derivatives (epidermis and neural tissue), a slightly lower incidence of differentiated mesodermal derivatives (cartilage and myotubes), and differentiated endodermal derivative (cylindrical epithelium; Fig. 3E). Surviving embryo‐derived teratomas treated with VPA were small, partially necrotic but upon histological assessment, some differentiated tissues were visible (Fig. 3C,D). In VPA‐treated teratomas, a statistically significant lower incidence of all differentiated tissues was discovered in comparison with controls (Fig. 3E). Only the mesenchyme (immature connective tissue) developed in all teratomas and was not influenced by VPA (Fig. 3E). Therefore, the potential for the development of tissues was diminished by VPA, especially for the neural tissue, but also for epidermis, cartilage, and cylindrical epithelium.

#### Valproate inhibits cell proliferation potential in embryo‐derived teratomas *in vitro*

**Figure 4:**
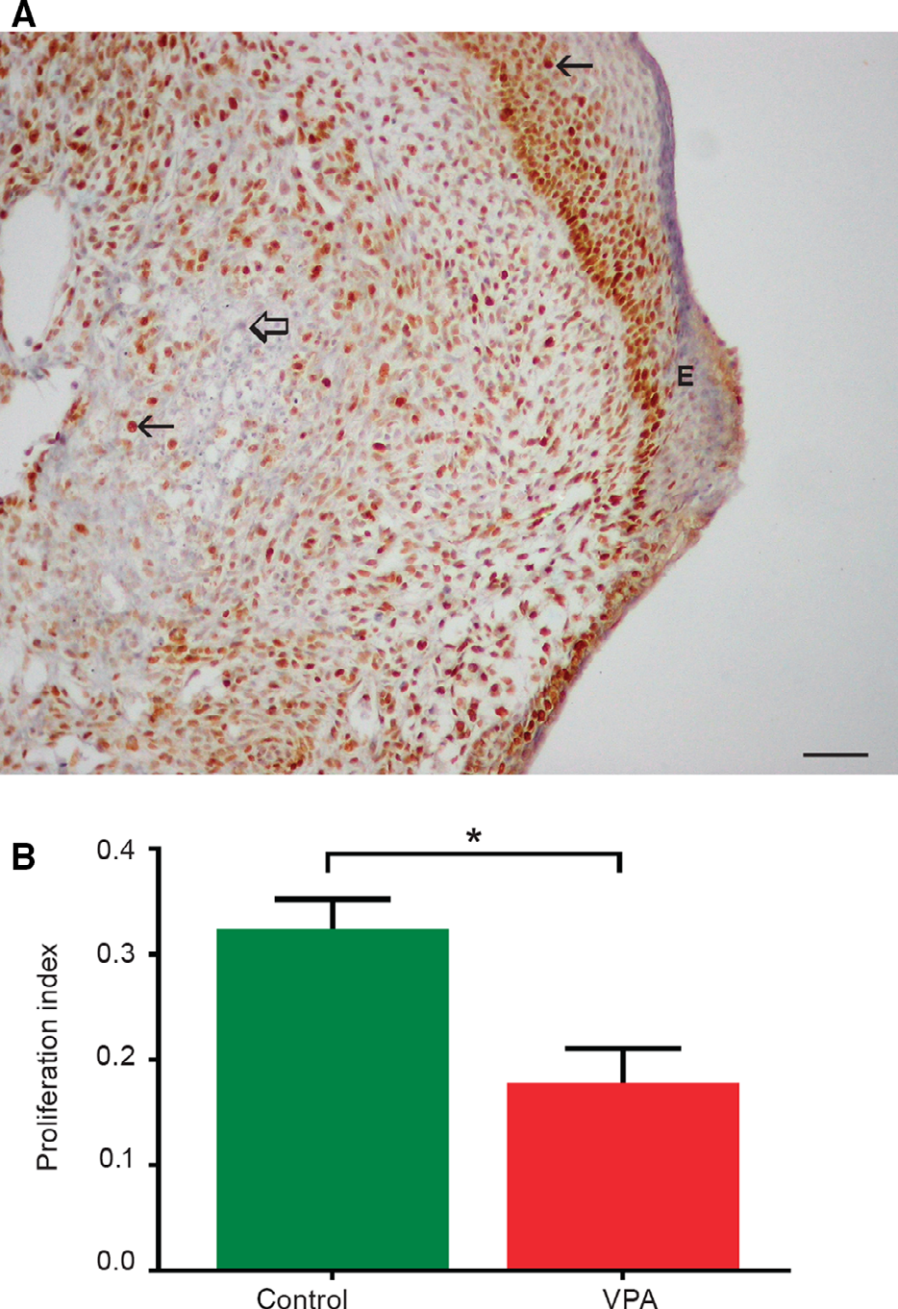
Expression of the PCNA in embryo‐derived teratomas cultivated for 14 days *in vitro*. (A) PCNA‐positive cells (arrows) in control teratomas; negative internal control (thick arrow), epidermis (E), scale bar = 50 μm. IHC, DAB, counterstained by hematoxylin. (B) Proliferation index (NPCNA/800 cells; *n* = 14 control embryo‐derived teratomas, *n* = 11 VPA‐treated embryo‐derived teratomas). Mean ± SEM. *P = 0.0055 (Student's *t*‐test, two‐tailed).

Because both the overall growth and differentiation of embryo‐derived teratomas were severely affected with 2 mm VPA, we were interested in whether VPA also influenced the potential for cell proliferation that is known to remain in controls at the end of the culture period [28]. The proliferation marker [proliferating cell nuclear antigen (PCNA)] was found in both VPA‐treated and untreated embryo‐derived teratomas after the 14 days of culture (Fig. 4A). The PCNA index was significantly lower in teratomas treated by VPA that confirmed the antiproliferative activity of VPA (Fig. 4B).

#### Valproate applied *in vitro* abolishes potential for further teratoma development *in vivo*

**Figure 5:**
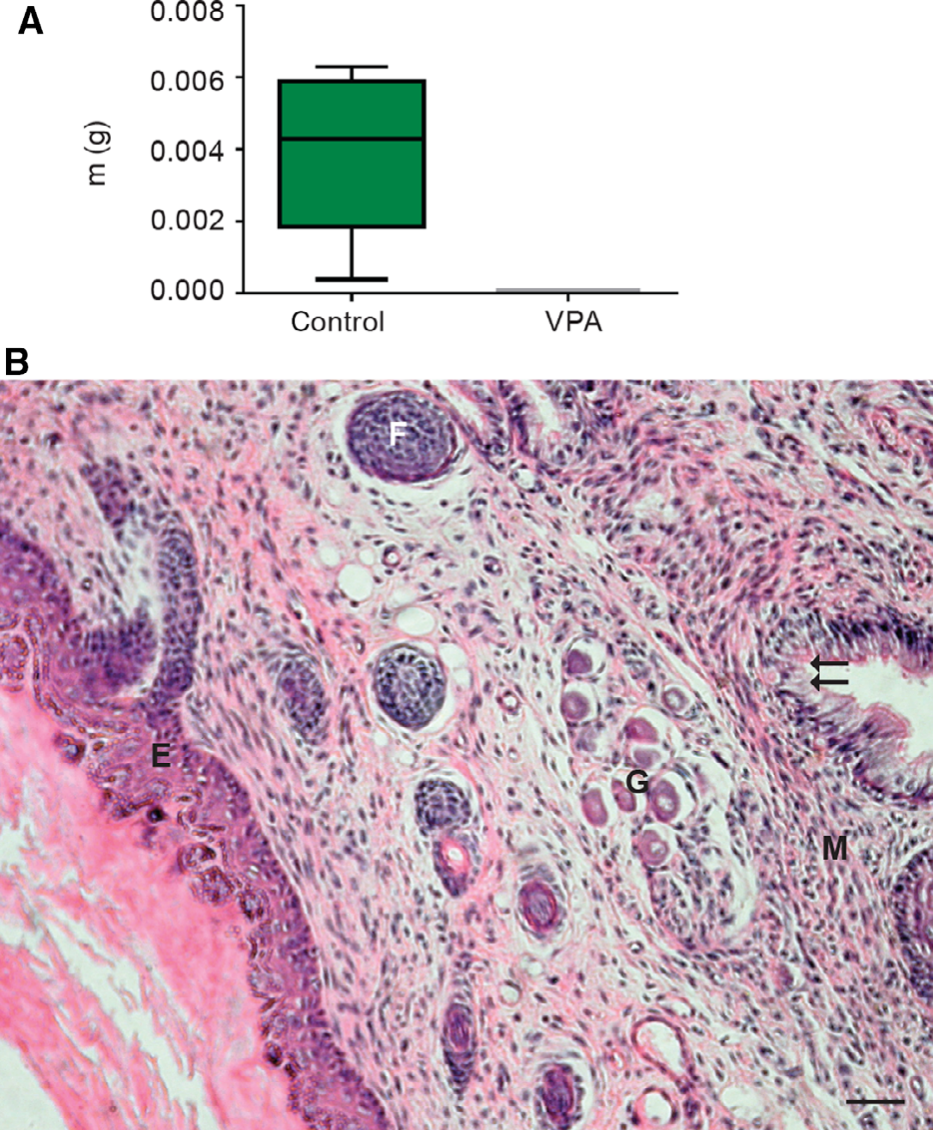
Isotransplants *in vivo* of embryo‐derived teratomas grown previously for 14 days *in vitro*. (A) Growth *in vivo* under the kidney capsule. Note that only controls were growing in transplants and were recovered for weighing and assessment of differentiation, while VPA‐treated teratomas did not develop at all. m(g) = mass in grams (*n* = 9 control embryo‐derived teratomas, *n* = 9 VPA‐treated embryo‐derived teratomas). Mean ± SEM. (B) Trilaminar differentiation of controls *in vivo*, the stratified squamous epithelium (E), the hair follicle (F), vegetative ganglion (G), muscle (M) pseudostratified columnar epithelium (arrows); scale bar = 50 μm, hematoxylin–eosin stain.

The enhanced apoptotic activity of developing embryo‐derived teratomas, necrosis, and diminished ability for cell proliferation after 14 days of culture caused by VPA prompted us to investigate whether they retained any potential for recovery and further development in a metabolically richer *in vivo* environment without VPA. All transplants of control embryo‐derived teratomas (nine samples) in this *in vivo* assay were able to grow and further differentiate under the kidney capsule for additional 14 days (Fig. 5A,B), developing, for example, epidermal appendages such as hair. In contrast to that, embryo‐derived teratomas pretreated with VPA *in vitro* (nine samples) resorbed, and only scars were found at the site of transplantation. This result shows that VPA was able to destroy all remaining potential for further development (growth and differentiation) *in vivo*.

#### Histone acetylation caused by valproate in cultivated embryo‐derived teratomas

**Figure 6:**
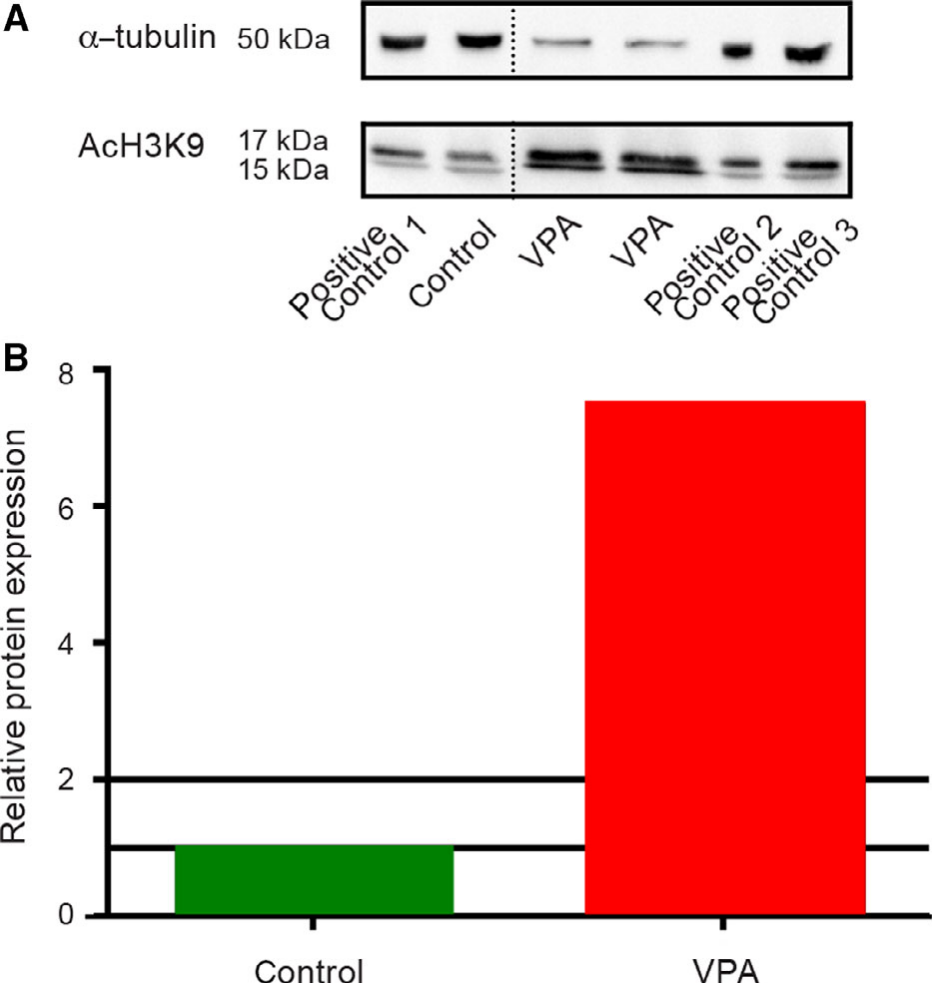
Histone H3 acetylation at the 14th day of culture detected by acetyl H3K9 antibody. (A) In control embryo‐derived teratomas (*n* = 16) and VPA‐treated embryo‐derived teratomas (*n* = 11), a positive signal for histone H3 acetylated at lysine 9 was detected by western blot [α‐tubulin (housekeeping protein); AcH3K9 = histone H3 acetylated at lysine 9; positive technical controls 1, 2, and 3]. The dotted line marks a splicing site where an intervening lane with a sample that was irrelevant for the analysis was removed. B Western blot analysis presented as change in the fold of global histone H3 acetylation in the VPA‐treated group normalized to the control group.

As VPA is an inhibitor of histone deacetylase, we wanted to confirm by western blotting (WB) that its deleterious effect on teratomas grown *in vitro* was associated with acetylation of histones. Results obtained by an appropriate antibody that targets lysine residues of histone 3 (H3AcK9) showed a significant 7.4871‐fold increase of histone H3 acetylation in the VPA‐treated group than in the control group of samples (Fig. 6) and confirmed the expected epigenetic influence of VPA.

### Spent media metabolomes

#### FTIR spectra of spent culture media metabolomes differ according to the treatment and periods of cultivation

**Figure 7:**
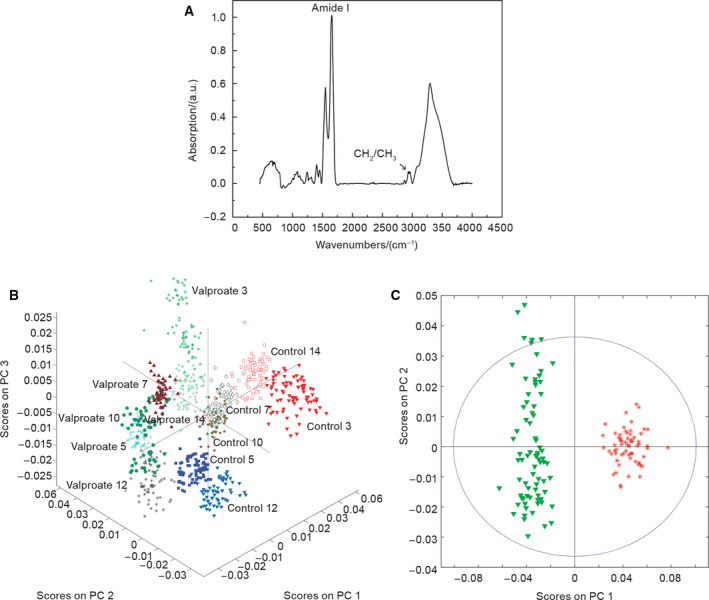
FTIR spectrum of a metabolome and the PCA. (A) Typical FTIR spectrum of a metabolome. The spectral ranges of interest were amide I band and CH_2_ and CH_3_ stretching vibrational bands (*n* = 6 groups of spent control media samples; *n* = 6 VPA‐supplemented groups of spent media samples). (B) Clustering of spectra according to the medium composition and the day of the culture when the spent medium was retrieved (3D PCA scatterplot). Scores on PC 1—16.94%, PC 2—10.55%, PC 3—5.60% (*n* = 6 groups of spent control media samples; *n* = 6 VPA‐supplemented groups of spent media samples). (C) 2D PCA scatterplot for all spectra retrieved from VPA‐treated teratoma (green) and control metabolomes (red) retrieved at the 3rd day of culture. Scores on PC 1—27.26%, PC 2—3.54%. (*n* = 6 groups of spent control media samples; *n* = 6 VPA‐supplemented groups of spent media samples).

In the embryo‐derived teratoma system, all developmental parameters described so far revealed the negative impact of VPA. We were interested in whether this activity could be reflected in metabolomes of spent cultivation media by using FTIR. Culture media were changed from the 3rd day on, and therefore, spent media were collected six times until the end of the culture period on day 14. Typical FTIR spectra of metabolomes and spectral ranges of interest are displayed in Fig. 7A. Principal component analysis (PCA) of FTIR spectra used to perform classification of samples showed that all metabolomes, either originating from different treatments or different days of culture, formed well‐separated clusters on a 3D PCA scores’ scatterplot (Fig. 7B). As an example of the general separation trend, 2D PCA scores’ scatterplot for control and VPA media after a 3‐day culture period is shown in Fig. 7C.

#### The dynamics of development of embryo‐derived teratomas is reflected in metabolomes

**Figure 8:**
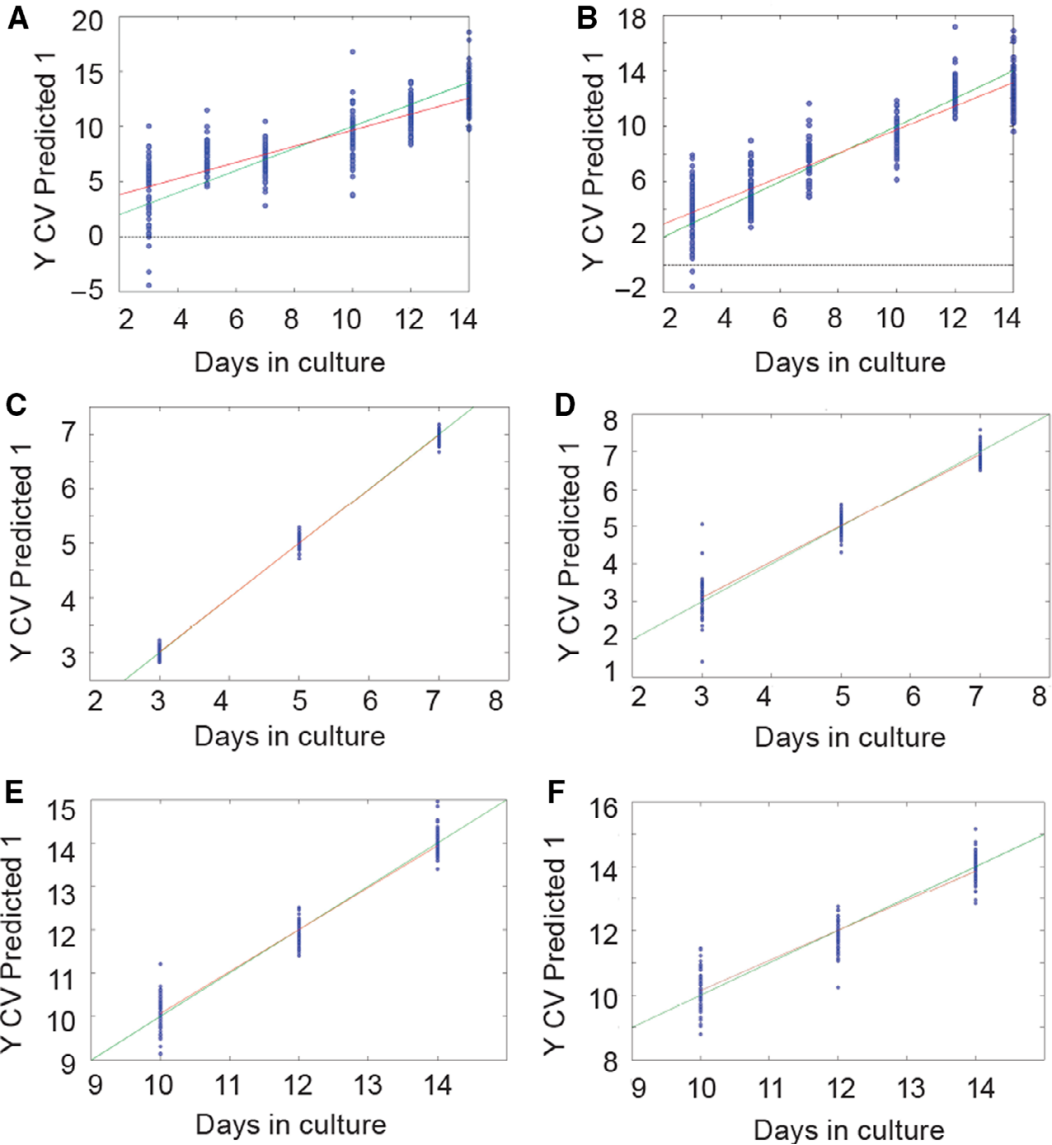
PC regression of FTIR spectra. The green line is representing an ideal correlation between measured and predicted values. The red line is representing the actual correlation. (A) Control teratoma metabolomes for the 3–14 days in culture (*R* ^2^ = 0.833, RMSEC = 1.3912, and RMSECV = 1.6297). (B) VPA‐treated teratoma metabolomes for the 3–14 days in culture (*R* ^2^ = 0.677, RMSEC = 1.971, and RMSECV = 2.2672). (C) Control teratoma metabolomes for the 3–7 days in culture (*R* ^2^ = 0.996, RMSEC = 0.088104, and RMSECV = 0.10383). (D) VPA‐treated teratoma metabolomes for the 3–7 days in culture (*R* ^2^ = 0.958; RMSEC = 0.24083; RMSECV = 0.33673). (E) Control metabolomes for the 10–14 days in culture (*R* ^2^ = 0.96 and RMSEC = 0.262833; RMSECV = 0.33235). (F) VPA‐treated teratoma metabolomes for the 10–14 days in culture (*R* ^2^ = 0.905; RMSEC = 0.42566; RMSECV = 0.51437). *n* = 6 groups of spent control media samples; *n* = 6 VPA‐supplemented groups of spent media samples.

In previous studies, dynamics of embryo‐derived teratoma development showed that the growth predominates during the first week of *in vitro* culture in the control group cultivated in the Eagle's essential medium with the rat serum, while differentiation is visible already from the second day on. Overt differentiation is mainly achieved during the second week when growth is subsiding [29, 30]. To estimate the possible variability/uniformity of spent media metabolomes that might correlate to developmental dynamics, we used principal component (PC) regression. We first analyzed metabolomes of control and VPA‐treated embryo‐derived teratomas obtained from the whole culture period, and then separately compared the metabolomes from the first and the second week (Fig. 8).

Principal component regression of FTIR spectra obtained for the whole culture period (days 3–14) showed a good correlation for both control and VPA‐treated groups (Fig. 8). Changes in the metabolome were less uniform for embryo‐derived teratoma samples cultivated with VPA in comparison with controls that might be related to the negative effects of VPA treatment.

For the control group during the first week of the culture (days 3–7), results of the PC (Fig. 8C,E) suggest that during the first week, while they predominantly grow, developing teratomas’ spent media metabolomes were more similar to each other than during the second week when teratomas definitively acquire various percentages of differentiated tissues (Fig. 3). The correlation coefficients for the first‐ and the second‐week metabolomes separately were higher than for the whole observed period. That is in concordance with the previous research, which showed the difference in dynamics of teratoma development between the 2 weeks [32].

For the VPA‐supplemented media, correlation coefficient for the whole culture period (days 3–14; Fig. 8B) was lower than the coefficient for the first week (days 3–7). Therefore, for embryo‐derived teratomas that were negatively affected by the VPA treatment, the variability of the metabolome change during the whole 2 weeks is higher than for the first week, only. Slightly higher variability was observed for the second week in comparison with the first week. These findings could relate to the VPA‐induced negative effects on development.

In conclusion, it may be said that the overall dynamics of embryo‐derived teratoma development is reflected in spent media metabolomes, especially when control and VPA‐treated metabolomes were compared.

#### Valproate increased intensities of FTIR vibrational bands specific for apoptosis and histone acetylation

**Figure 9:**
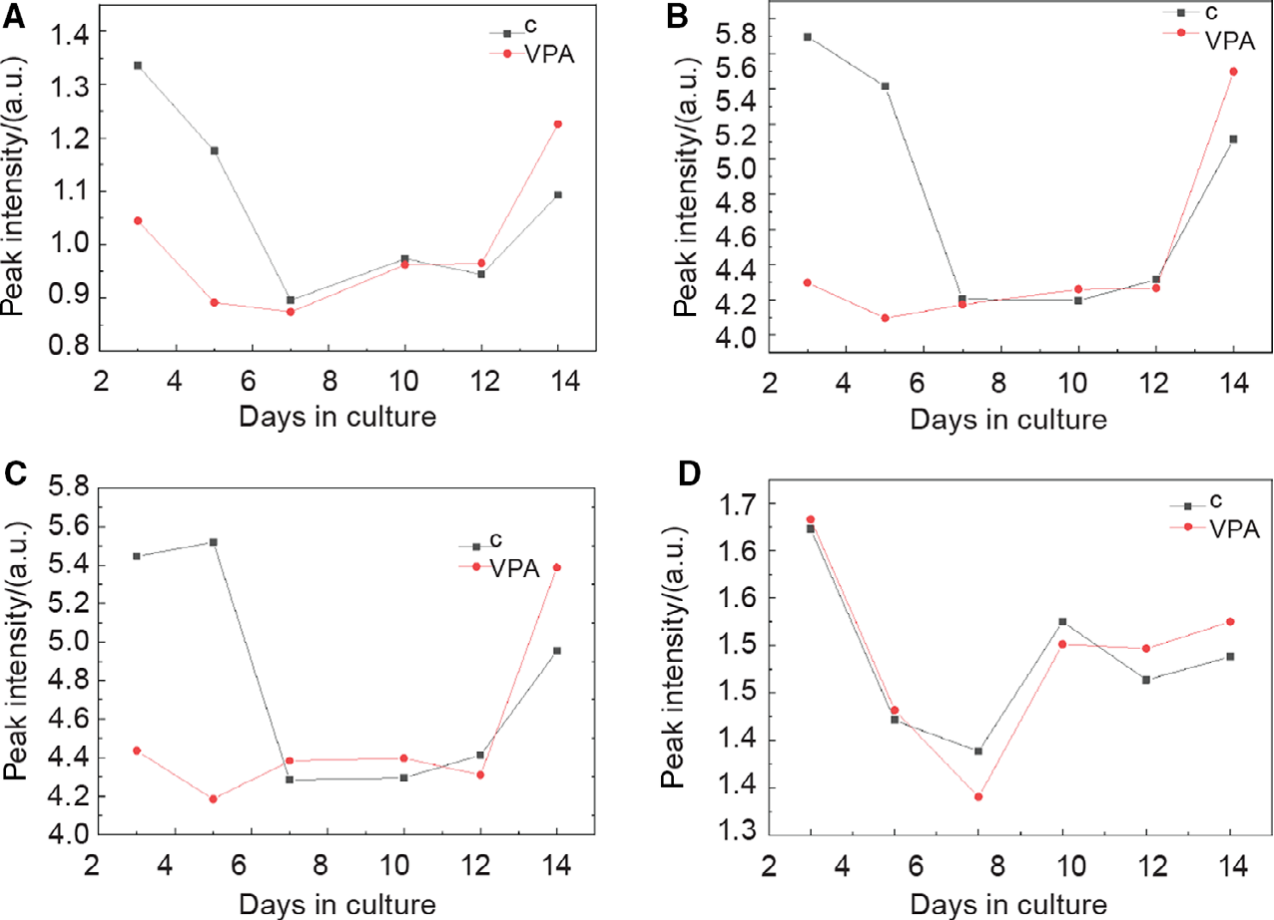
Fitting results for peak intensity of four amide I protein vibrational bands. (A) β‐sheets at 1625 cm^−1^. (B) Unordered at 1641 cm^−1^. (C) α‐helix at 1660 cm^−1^. (D) Turn at 1683 cm^−1^. Black—control group, red—VPA‐supplemented group; *n* = 6 groups of spent control media samples collected on different days of culture; *n* = 6 VPA‐supplemented groups of spent media samples collected on different days of culture.

**Figure 10:**
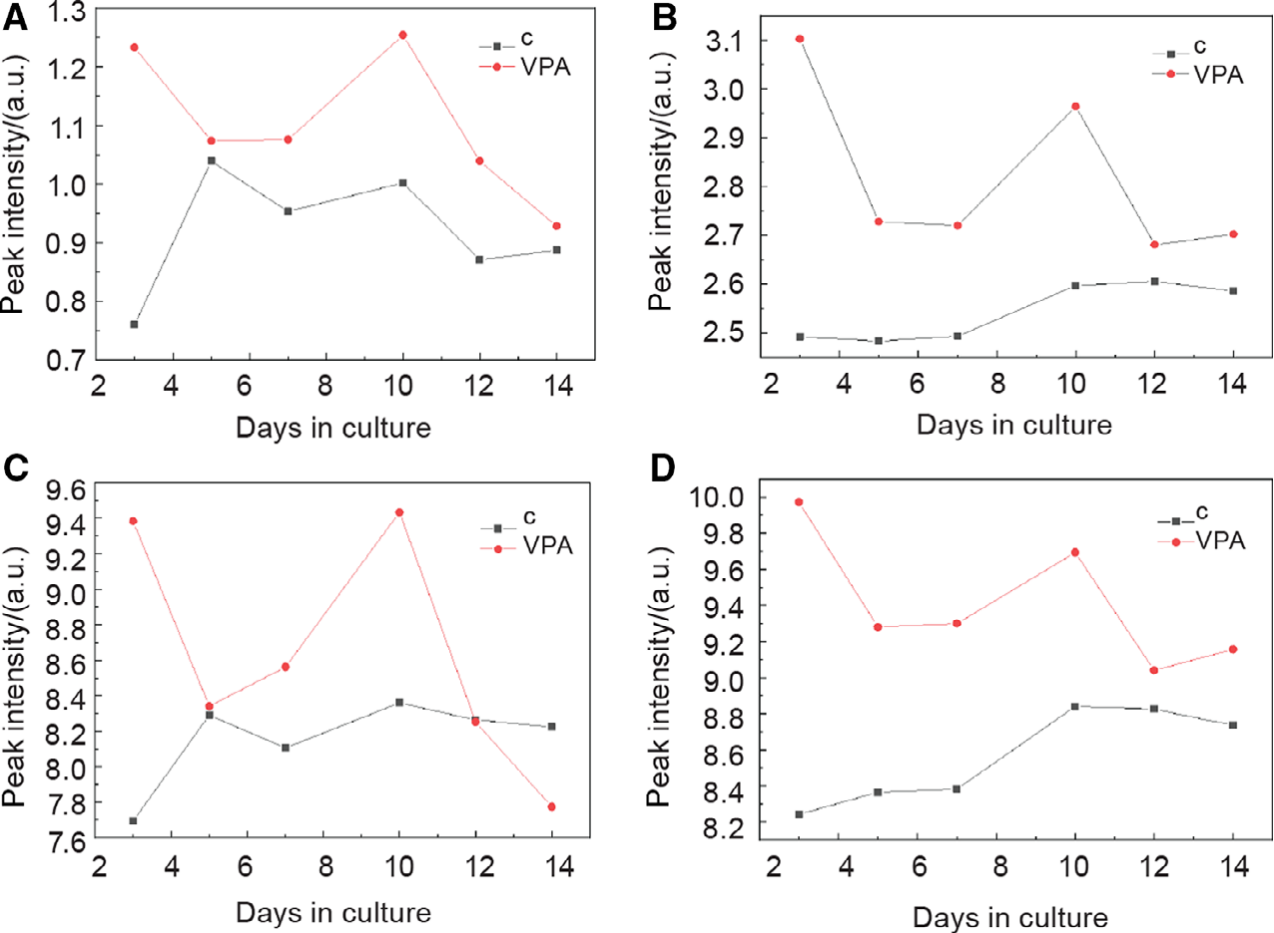
Fitting results for peak intensity of four CH stretching vibrational bands. A 2853 cm^−1^ for CH_2_ vibrations of lipids (symmetric stretching modes). B 2874 cm^−1^ for CH_3_ vibrations of lipids and proteins. (C) 2924 cm^−1^ for CH_2_ vibrations of lipids (asymmetric stretching modes). (D) 2960 cm^−1^ for CH_3_ vibrations of lipids and proteins (black—control group of spent media, red—VPA‐supplemented group of spent media). *n* = 6 groups of spent control media samples; *n* = 6 VPA‐supplemented groups of spent media samples.

**Figure 11:**
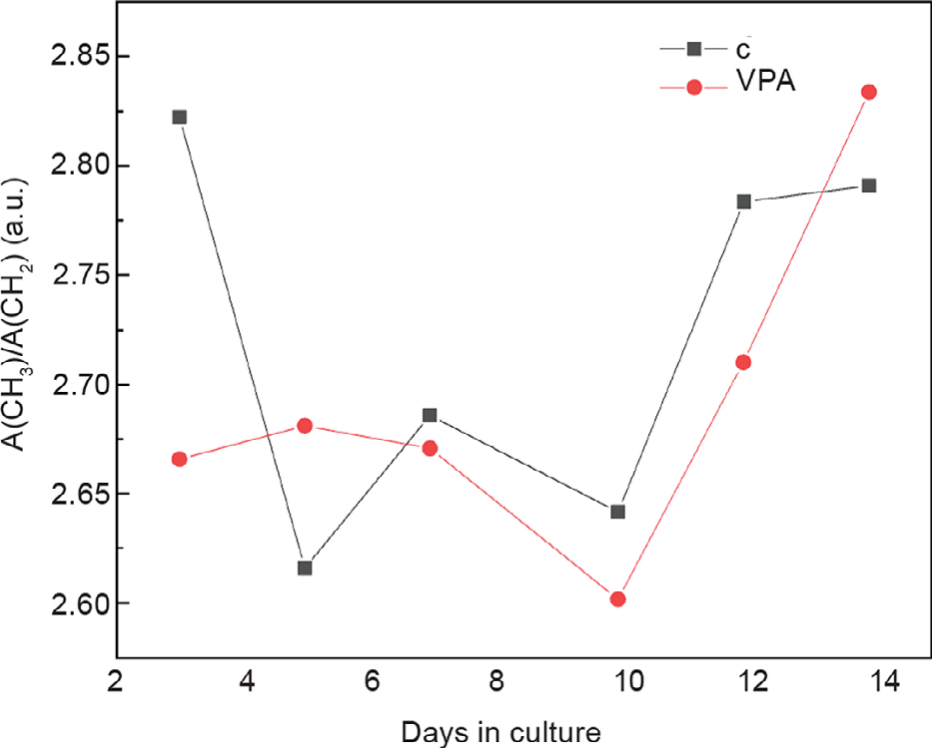
Change of stretching vibration intensity ratio A(CH_3_)/A(CH_2_) with the cultivation time. Black—control group, red—VPA‐supplemented group. *n* = 6 groups of spent control media samples; *n* = 6 VPA‐supplemented groups of spent media samples.

Since we confirmed histone acetylation (Fig. 6) and apoptosis (Fig. 2) in embryo‐derived teratomas treated with VPA, we tried to assess histone acetylation and apoptotic markers possibly released to their spent media. Based on previous research of FTIR biomarkers associated with histone acetylation and apoptosis [51, 52, 53], we calculated relevant intensities of four amide I vibrational bands and vibrational bands related to the secondary structure of proteins and lipid absorbance (Figs [9, 10, 11]).

#### Amide I vibrational bands

For the average intensities of the four relevant FTIR vibrational bands that compose the amide I range of proteins according to Zhang *et al*. [51], spectral range for different days of the whole culture period (3–14 days) was assessed by curve fitting of all recorded spectra followed by averaging the obtained results for observed groups of samples.

As seen in Fig. 9, for days 3 and 5 metabolomes from the first cultivation week, the intensities of amide I β‐sheet (at 1625 cm^−1^; Fig. 9A), unordered amide I (at 1641 cm^−1^; Fig. 9B), and α‐helix (at 1660 cm^−1^; Fig. 9C) vibrational bands of metabolome influenced by VPA were first lower than in the control group. From day 7, the difference significantly decreased, and on day 14, the average intensities in VPA‐treated metabolomes exceeded those in controls. For the turns in the amide I (at 1683 cm^−1^; Fig. 9D), the difference was almost nonexistent for days 3 and 5. At day 7, the intensities of VPA‐treated metabolomes started to rise, and on day 14, the intensities in spectra of VPA‐treated metabolomes were higher.

Therefore, the intensities of all VPA‐treated teratomas for amide I vibrational band were higher than in the control group at the end of the culture.

#### Lipid vibrational bands

The second range of vibrational spectra that we analyzed consisted of bands from 2853 to 2960 cm^−1^, mainly assigned to lipids, according to Zhang *et al*. [51] (Fig. 10). All spectra intensities in metabolomes of VPA‐treated teratomas were higher than the intensities of the control group throughout the whole culture period except for the band at 2924 cm^−1^ for CH_2_ vibrations of lipids (asymmetric stretching modes; Fig. 10C).

Next, we found out that the band ratio of [I(2960) + I(2937) + I(2874)]/[I(2924) + I(2853)] cm^−1^ (i.e., the ratio of the stretching vibration intensity), namely, A(CH_3_)/A(CH_2_), increased at the end of culture period for VPA‐treated teratomas (Fig. 11).

The above results from VPA‐treated embryo‐derived teratoma metabolomes in comparison with controls are in concordance with previous investigations of FTIR biomarkers that associate similar findings with the higher protein acetylation levels [51] and apoptosis [52, 53], as discussed later. Therefore, we may conclude that metabolomes of VPA‐treated teratomas reflected both histone acetylation and apoptosis that VPA caused in embryo‐derived teratomas.

## Discussion

### The deleterious impact of VPA on the embryo‐derived teratoma *in vitro*

Although the antitumor activity of VPA has been under investigation for human tumors other than teratoma [7, 54, 55, 56], we now report that VPA exerted a deleterious and, therefore, a therapeutic effect on a teratoma‐type tumor *in vitro* affecting exactly the undifferentiated and poorly differentiated source of the tumor. VPA activity affected all relevant parameters that histopathologically characterize targets for an antitumor therapy because, during cultivation, VPA impaired the survival, growth, and cell proliferation, and enhanced apoptosis and necrosis. Moreover, VPA inhibited possible recovery and further growth of teratomas in the VPA‐free ectopic site *in vivo* under the kidney capsule that is providing an optimal blood supply able to support teratoma development even from the very small transplants such as single germ layers [15].

Based on this *in vitro* therapeutic effect that acted on the undifferentiated component, we may suppose that other forms of teratoma tumors such as the GTS [22] or teratocarcinoma/EC that present a serious problem in young males [19, 24] might also be responsive to the VPA therapy because in the *in vitro* system VPA is acting already at the beginning of the culture period characterized by the intensive proliferation of undifferentiated primitive streak and the three germ layer cells that might be the source of such tumors [19]. This has to be corroborated by future *in vivo* experiments.

As expected for a HDACi, we confirmed that VPA‐induced histone acetylation took place in embryo‐derived teratomas, but the negative effect exerted by VPA might have been caused additionally by acetylation at the lysine of other important proteins involved in proliferation [57], differentiation, and apoptosis among which the products of oncogenes or tumor‐suppressor genes proposed as cancer therapy targets [58]. Indeed, over a hundred differentially expressed lysine acetylation sites (Kacs) were found in acetylome as the result of the antitumor therapy [59]. Some of those Kacs were acetylated by the HDACis suberoylanilidehydroxamic acid (SAHA) and VPA, although their impacts on the acetylome were different [60].

Lysine acetylation and deacetylation were associated, for example, with brain development [04], while in the embryo‐derived teratoma system, VPA severely affected differentiation of all germ layer derivatives, among which also the neural tissue. Although VPA is a known teratogen that specifically influences brain development [61], basic research in various systems on the mechanisms of its teratogenicity is still necessary [62]. Important research on neural development is conducted in the gastrulating whole rat embryo (WEC) *in vitro* system that includes extraembryonic membranes and is of a shorter duration than this system [63], as well as in cultivated neuronal embryonic stem cells [58] first derived from the inner cell mass of the blastocyst.

Transcriptomic evaluation of VPA activity across *in vitro* developmental models (WEC, cardiac, and neural embryonic stem cells) and nondevelopmental models has shown that developmental models were better for assessment of developmental‐specific effects such as for neuronal differentiation [64]. Because this *in vitro* developmental model affecting the gastrulating embryo without any extraembryonic parts clearly showed that neural differentiation was inhibited by VPA, we may state that the direct impact on the embryo‐proper was assessed without the confounding effects of extraembryonic tissues. Moreover, we are dealing with a natural 3D biological system where some tissue interactions are still possible. Therefore, this model may be used for further screening of other HDACis or different dosages of VPA, as well as for other developmental hazards influencing the embryo itself. As the gastrulating human embryo is still not accessible for research [65, 66], only the rodent embryos may be investigated at this most critical phase of mammalian development in defined *in vitro* systems such as this [19, 28].

### Specific changes in metabolomes reflect VPA activity in embryo‐derived teratomas

In the embryo‐derived teratoma system, FTIR analysis enabled us to distinguish all metabolomes according to the type of spent medium or the day/week of culture (Figs [7 and [8), thus successfully associating developmental and metabolome dynamics by an integrated experimental and computational approach important for a true system‐level approach [43].

#### Histone acetylation

In accordance with the acetylation of proteins that we assessed on the histones in embryo‐derived teratomas themselves by WB (Fig. 6), FTIR analysis revealed an elevated level of the amide I α‐helices (Fig. 9) and an elevated ratio of A(CH_3_)/A(CH_2_), a ratio of the stretching vibration intensity from CH_3_ methyl to CH_2_ methylene (Fig. 11) in the same way as described by Zhang *et al*. [51], dealing with HeLa cells treated with another HDACi, trichostatin A. Indeed, that histone acetylation specifically increases the α‐helical content of histone tails was experimentally confirmed also by other methods [67].

Valproate increased the intensity of four CH stretching vibrational bands mostly assigned to lipids in metabolomes of embryo‐derived teratomas (Fig. 10). The lysine acetylation of enzymes involved in metabolic processes among which those for fatty acid synthesis were found in acetylomes of evolutionary divergent species [60, 68, 69]. We may presume that an increase of lipids that we detected may be the consequence of enhanced lipid synthesis, but that must be proven by future experiments.

#### Apoptosis and necrosis

In these experiments, amide I components of proteins (secondary structures) were all of higher intensity in VPA‐treated metabolomes in comparison with controls at the end of the culture period (day 14 metabolomes; Fig. 10). It was reported in 2009 by Zelig *et al*. that the absorption band for the parallel β‐strand structure, by a dramatical concentration‐dependent increase, also characterized apoptosis in U937 cells treated with high doses of cytosine arabinoside chemotherapeutic [52]. Liu *et al*. in 2001 [53] found a shift of β‐strand structure to unordered amide I as the percentage of apoptosis increased. We may presume that in the metabolomes, both β‐strand structure and unordered amide I absorption bands reflected apoptosis induced in teratomas by VPA. Although VPA has induced apoptosis already on the third day of culture (Fig. 2), it is possible that both structures are first released from the tissue into the medium upon the final decay of teratomas at day 14 of culture.

CH_2_ vibrations of lipids that were increased in metabolomes of teratomas treated with the VPA already from the day 3 to the day 14 of culture (Fig. 10) previously were also linked to apoptosis [52, 53, 70], although Zelig *et al*. [52] state that changes in lipid absorbance cannot be used to distinguish between apoptosis and necrosis. Lipids seem to be released from embryo‐derived teratomas already on the third day and, therefore, may present an early marker of cell death in the metabolome [52].

In 2009, Zelig *et al*. [52] found a decrease in the random coil of amide I in defining necrosis of cells caused by KCN. We also found the same in control metabolomes already at day 7 as well as a low level in VPA‐supplemented medium from day 3 to day 12, although afterward, the random coil of amide I level was higher. It is possible that this marker reflects necrosis also in the metabolomes.

Finally, investigation of spent media metabolomes in the embryo‐derived teratoma system did reflect not only the difference in treatments (VPA versus control) and the developmental dynamics of the *in vitro* development but also reflected acetylation of histones/proteins and apoptosis found in teratomas through specific FTIR spectral markers.

## Conclusion

We have been able to prove that the lysine acetylation activity of VPA exerts a deleterious effect on the development of the embryo‐derived teratoma *in vitro* that may suggest a novel strategy in the therapy of teratoma tumors *in vivo*. By analyzing metabolomes from the spent media by FTIR spectroscopy that we associated with the developmental dynamics and biomarkers of histone acetylation and apoptosis, we completed the biological system. These findings lead us to the conclusion that the embryo‐derived teratoma system, completed by the rapid FTIR spectroscopy, represents a robust, fast dual *in vitro* screening biological system for the antitumor and embryotoxic/teratogenic agents.

## Materials and methods

### Ethical statement

All animal procedures were conducted according to the Directive 2010/63/EU and those of Croatian Law on the protection of experimental animals. They were approved by the Ethical Committee of the School of Medicine, University of Zagreb, Croatia.

### Animals

Fisher strain inbred rats were obtained from the registered animal facility for laboratory rodents at the School of Medicine, University of Zagreb, Department of Medical Biology.

Three‐month‐old dams and males were kept in conventional cages, with standard diet and bedding with GLP certificate and water *ad libitum* at 20–24 °C, relative humidity 40–70%, day/night cycle 12/12 h, and a noise level under 60 dB.

Males and females were caged together overnight, and if the next morning sperm was found in the vaginal smear, the noon was declared as the 0.5 day postcoitus (dpc). At 9.5 dpc, rat dams were anesthetized with 0.8 mL·kg^−1^ of ketamine (Narketan®; Vétoquinol, Bern, Switzerland) and 0.6 mL·kg^−1^ of xylazine (Xylapan®; Vétoquinol). Deciduae were removed from the uteri, and under a dissecting microscope, egg cylinders were isolated with the watchmaker's forceps. Reichert's membranes and ectoplacental cones were removed, and the extraembryonic portion cut at the level of the amnion to isolate the gastrulating embryo‐proper consisting of the three germ layers only.

### *In vitro* culture

Three embryos‐proper were plated on a lens paper supported by a stainless steel grid in a 60 × 15 mm center well organ culture dish (BD Falcon™, Oxford, UK) with enough medium in the well to wet the lens paper. Teratomas were grown either in Eagle's minimum essential medium (MEM) with Hank's balanced salt solution supplemented with 50% rat serum at the air–liquid interface to serve as controls or in the same medium with 2 mm VPA (#P4543; Sigma, St. Louis, MO, USA). Blood of anesthetized male rats with 0.8 mL·kg^−1^ of ketamine (Narketan®; Vétoquinol) and 0.6 mL·kg^−1^ of xylazine (Xylapan®; Vétoquinol) of the same strain was drawn and heat‐inactivated [64, 71]. Teratomas spent 2 weeks in an incubator at 37 °C in 5% CO_2_ and 95% humidified air. After plating, media were changed five times from the 3rd day of culture on. Altogether, 12 spent media from all organ tissue dishes accommodating 37 control and 38 experimental samples were collected, pooled according to the treatment and the day of the culture (3rd–14th day), and frozen at −80 °C.

### Survival

Survival was finally established in 14‐day‐old samples by light microscopy. Samples that disappeared during cultivation or were completely necrotic did not survive [72].

### Overall growth

The overall growth of ellipsoid teratomas was noninvasively measured (14×) throughout the 2‐week culture period. The major and minor diameters were measured using an ocular micrometer, which was shown before to correlate with the DNA and RNA and protein concentration [29], and the ellipse area was calculated (*A* = π × major diameter × minor diameter/4). All values were normalized by dividing with values of the initial measurement at plating and used as the measure of overall growth (*A*/*A*_0_) [28, 73]. Therefore, *A*/*A*_*0*_ was 1 for day 0 when embryos were first plated.

### Isotransplants *in vivo*

Teratomas cultivated *in vitro* for 14 days were subsequently transplanted to an ectopic site under the kidney capsule of adult male Fisher rats. Rats were anesthetized with 0.8 mL·kg^−1^ of ketamine (Narketan®; Vétoquinol) and 0.6 mL·kg^−1^ of xylazine (Xylapan®; Vétoquinol), and the kidney was approached through a paravertebral incision. A small pocket was formed under the kidney capsule with a Graeffe's knife, and a fine forceps and teratomas were transferred using a braking pipette. Sixteen millimeter Michel's clamps were used to close the wound. Isotransplants were grown *in vivo* for another 2 weeks.

### Histology and immunohistochemistry

Both *in vitro* and *in vivo* grown teratomas were fixed for 24 h in mild St. Marie solution (1% glacial acetic acid in 96% ethanol), dehydrated, and embedded in paraffin. Serial sections (5 μm) of a single teratoma were processed for routine histology or IHC.

For histological analysis of survival and differentiation, hematoxylin‐/eosin‐stained sections were used as previously described [28, 74]. Survival was calculated as the number of teratomas containing recognizable cells that were present at the end of the 14‐day culture period, while the incidence of differentiated tissue was assessed in the control or experimental group of teratomas and expressed as percentage of the number of teratomas.

For immunohistochemical labeling, microscopic slides were deparaffinized, cleared in xylene, and hydrated to TBS in graded alcohol solutions. Antigen retrieval was performed in Dako retrieval buffer pH = 6.0 in a microwave oven on 700 W. After boiling, slides were cooled for 1 min, a treatment repeated three times. Slides were then cooled for 20 min, blocked with peroxidase blocking reagent (0.03% H_2_O_2_) for 20 min, and rinsed three times in TBS. Primary antibodies were diluted in 1% BSA/TBS/0.05 Tween‐20 and applied on sections overnight at 4 °C. Primary antibodies used in this study were mouse anti‐human PCNA monoclonal antibody (Clone PC‐10, M0879; Dako, Glostrup, Denmark), 1 : 100, and cleaved caspase‐3 monoclonal antibody (#9661; Cell Signaling Technology, Inc., Danvers, MA, USA), 1 : 200. On the second day, slides were washed five times for 5 min with TBS and incubated for 45 min with Dako Dual Link HRP‐conjugated secondary antibody (K4063, Dako). Visualization of the signal was done with DAB (3,3′‐diaminobenzidine) and chromogen–substrate complex for 1 min and stopped in dH2O. Slides were counterstained with hematoxylin, washed with tap water for 20 min, and covered with glycerol/TBS solution (1 : 1).

### Stereology

Anti‐cleaved caspase‐3 signals were stereologically quantified using the volume density (*V*_v_) in control and experimental teratomas. The analysis was performed using the Nikon Alphaphot binocular light microscope (Nikon, Vienna, Austria) with Weibel's M42 test system, made of 42 short test lines, each with two ends as test points [73]. Volume density (*V*_v_) was estimated by counting points of the test system, which hit stained nuclei and points which hit the reference space at 400× magnification (reference space being defined as hits on any part of a section) and calculated as the ratio between the hits falling in all stained nuclei (Pi) and hits falling in the reference space (Pt; *V*_v _= Pi/Pt), and expressed in mm^0^ (mm^3^/mm^3^). The stereological orientation measurement was carried out to define the number of fields to be tested [73]. At least 90 fields were assessed.

### Proliferation index

For analyzing the PCNA expression, six teratoma samples per group were randomly chosen, serially sectioned, and for each sample, four nonadjacent sections from different regions were scored in 800 cells at 1000× magnification. Every DAB‐stained (brown) nucleus was considered as positive, irrespective of the staining intensity. The proliferation index was calculated as the number of PCNA‐positive cells/800 cells [73, 75].

### Tissue homogenization

Sixteen control and 11 samples from the VPA‐treated group of teratomas were pooled due to the very small protein content in each experimental teratoma. They were placed in two separate collection tubes containing 100 μL of RIPA buffer [Tris/HCl pH 8.0 50 mm, NaCl 150 mm, SDS 0.1%, Na deoxycholate 0.5%, Triton X‐100 1%, 1% 0.5 m EDTA, and 4% complete EDTA‐free protease inhibitor (COEDTAF‐RO Roche, Sigma Aldrich, Germany)] and 10 sterile glass beads 1.0–1.51 mm in diameter (Retsch, Haan, Germany). Tissue was homogenized by a bead‐based homogenizer (Bertin) for 2 min at 5000 r.p.m.

### SDS electrophoresis

Protein concentration was detected by bicinchoninic acid assay (BCA; Sigma, BCA1) using an Uvikon‐860 spectrophotometer (Kontron Instruments, Zurich, Switzerland ), according to the manufacturer's instructions. The tissue lysate was mixed with ¼ Laemmli buffer (Tris/HCl pH 6.8 0.125 m, glycerol 20%, 2‐mercaptoethanol 10%, SDS 4%, bromophenol blue 0.004%) boiled for 5 min and centrifuged at 16 000 ***g*** for 1 min. Ten microgram of protein was loaded per well of the gel and run in the Mini‐PROTEAN Tetra cell system (Bio‐Rad) alongside a protein marker (1610375, Precision Plus Protein™ Kaleidoscope™ Standards; Bio‐Rad Laboratories, Hercules, CA, USA). Buffer formulations and run times were used according to the General protocol for WB by Bio‐Rad.

### Western blot

Blots were prepared using a Mini Trans‐Blot Cell (Bio‐Rad, 1660828EDU) and PVDF Immobilon membrane (Millipore, Bedford, MA, USA) using buffer formulations and run times according to the General protocol for WB by Bio‐Rad.

The membrane was blocked for 1 h in 3% BSA in TBST (20 mm pH 7.5 Tris/HCl, 150 mm NaCl, and 0.1% Tween‐20), rinsed with TBST, and incubated overnight at 4 °C with primary antibodies to histone H3 acetyl K9 (ab10812, dilution 1 : 500 in 3% BSA/TBST Abcam, Cambridge, MA, USA) and α‐tubulin (Abcam, ab52866, dilution 1 : 10 000 in 3% BSA/TBST). The membranes were rinsed with TBST and then incubated with secondary antibody goat anti‐rabbit (Abcam, ab97051, dilution 1 : 20 000 in 3% BSA/TBST) for 1 h at room temperature. The membranes were rinsed and treated with a chemiluminescent (Immobilon Western; Millipore) using ChemiDoc XRS+ (Bio‐Rad). The signal was quantified using image lab™ 6.0 (Bio‐Rad). The obtained signal for histone H3 (acetyl K9) was normalized to the α‐tubulin signal as the housekeeping protein for each group, respectively. Obtained values for the VPA‐treated group were further normalized to the control values [76]. Fold minor to 0.5 and major than 2.0 was defined as significant. For technical western blot positive controls, indifferent tissue was used.

### Statistical analysis for *in vitro* embryo‐derived teratoma

Descriptive analysis was made for all experiments, and the normal distribution of the data was tested, after which parametric or nonparametric tests were chosen to compare variances.

The overall growth of teratomas was tested by the Mann–Whitney test, and for survival and differentiation analysis, proportions of surviving teratomas or differentiated tissues in experimental teratomas were compared by chi‐square or Fisher's exact test. Differences in the volume density (*V*_v_) of anti‐cleaved caspase‐3‐positive cells were tested using the Mann–Whitney test and proliferation index by Student's *t*‐test. The statistical significance level was set at *P* < 0.05.

### FTIR sample preparation

Spent media samples were defrosted at room temperature for 30 min, and 5 μL of each sample was transferred to an optical grade silicon plate. To remove any interference from vibration and rotation of water molecules, plates were dehydrated under vacuum for 5 min. The samples were placed into the sample compartment of the spectrometer for IR transmission measurements.

### Sample analysis by infrared spectroscopy

Measurements were performed using a spectrometer (PerkinElmer GX, Inc., Waltham, MA, USA), equipped with an IR source, emitting between 10 and 15 600 cm^−1^. An MCT detector, cooled with liquid nitrogen, was used for scanning. Background spectra were excluded from the optical plate itself, by measuring and automatically subtracting from the scanned sample spectra. Twenty spectra were recorded for each control and VPA‐supplemented spent media sample in the spectral region between 450 and 4000 cm^−1^. A background spectrum was recorded from 500 scans, and for the sample spectra, 250 scans were recorded and averaged at a spectral resolution of 4 cm^−1^ in a transmission mode. At least 800 different spectra were recorded and stored electronically for further statistical analysis[77, 78].

### Statistical analysis for FTIR

Multivariate statistical methods were used to perform the classification of samples: PCA and PC regression using cross‐validation. The PCA method was used to efficiently reduce the data set to a few that give the highest variability among the recorded spectra. When spectra were analyzed in this low‐dimensional PCA space, cluster formation with a similar metabolic ‘fingerprint’ was notable. Statistical analysis was performed using matlab (MathWorks, Natick, MA, USA) with PLS‐Toolbox (Eigenvector Research, Inc.; Wenatchee, WA, USA). PC regression analysis was used to compare the regression coefficient (*R* ^2^), a root mean square error of calibration (RMSEC), and root mean square error of cross‐validation (RMSECV). The best results are obtained with *R* ^2^ being close to 1 and RMSEC and RMSECV being close to 0.

## Conflict of interest

The authors report no conflict of interest. The authors alone are responsible for the content and writing of the study.

## Author contributions

MP designed the research, conducted experiments, and interpreted results; AKB designed, supervised, and conducted histological assessments and statistical analysis; MHP conducted histological analysis; OG designed and conducted analysis and interpretation of FTIR results; MR conducted FTIR experiments and analysis; VR conducted FTIR experiments and analysis; ŠM conducted FTIR experiments and interpreted FTIR results; MK conducted FTIR experiments and interpreted FTIR results; JK conducted western blot analysis; NS designed and conducted *in vivo* experiments and designed western blot analysis; GJ‐L conducted histological analysis and interpretation of results; MB† designed, supervised FTIR research, and interpreted the results; FB‐J designed, supervised the research, and interpreted the results. All authors participated in writing the article.
